# Learning of Basic Life Support through the Flipped Classroom in Secondary Schoolchildren: A Quasi-Experimental Study with 12-Month Follow-Up

**DOI:** 10.3390/medicina59091526

**Published:** 2023-08-23

**Authors:** Miguel Cons-Ferreiro, Marcos Mecias-Calvo, Vicente Romo-Perez, Rubén Navarro-Patón

**Affiliations:** 1 Faculty of Education and Sport Sciences, Campus a Xunqueira, s/n, Universidade de Vigo, 36005 Pontevedra, Spain; miguelcons@uvigo.es (M.C.-F.); vicente@uvigo.es (V.R.-P.); 2 Facultad de Formación del Profesorado, Universidade de Santiago de Compostela, 27001 Lugo, Spain; ruben.navarro.paton@usc.es

**Keywords:** schoolchildren, skills, cardiopulmonary resuscitation, automated external defibrillator, training method

## Abstract

*Background and Objectives*: International institutions together with the World Health Organisation recommend the teaching of BLS in schools. Therefore, the objective of this research was to study the feasibility of teaching CPR and AED through the flipped classroom, exploring the medium- and long-term retention of knowledge and practical skills among high school students. *Materials and Methods*: The sample consisted of 260 secondary schoolchildren (137 in the experimental group (EG) and 123 in the control group (CG)) between 12 and 14 years old (*M* = 12.75 ± 1.02). *Results*: The data revealed that the EG obtained better post-course results in the correct position of the hands (*p* = 0.011), the depth of external cardiac compression (*p >* 0.001), and the mean time to apply an effective shock with the AED (*p* = 0.013). The CG obtained better results in compressions with complete chest re-expansion (*p* = 0.025). These differences disappeared at 6 months (*p* > 0.05) and 12 months (*p* > 0.05). *Conclusions*: A training program based on the flipped classroom is as effective and viable as traditional training, although more efficient since it is applied in less time, in the sequence of action in BLS, CPR skills, and the application of an effective shock with an AED.

## 1. Introduction

Training in basic life support (BLS) and using an automated external defibrillator (AED) of the general population is widely recognised to promote a positive attitude towards cardiopulmonary resuscitation (CPR) [1]. Additionally, bystander BLS improves survival chances in out-of-hospital sudden cardiac arrest [2].

CPR and AED training in schools is considered an essential and effective part of disseminating knowledge, skills, and positive attitudes towards CPR and AED among the general population [3,4,5] since it is an easily accessible population that is attracted to learning these contents and skills [6,7]. For this reason, education in BLS in early childhood can be key not only because it can increase the effective number of witnesses who provide cardiopulmonary resuscitation (CPR) and early defibrillation [8], but also because schoolchildren are considered potential knowledge multipliers in BLS [9,10,11].

It has long been established that BLS and AED courses improve knowledge immediately after application, while knowledge and skill retention have been explored less [6,12]. Previous studies indicate that CPR skills can deteriorate in 3–6 months [5,13,14], while AED use skills last longer [13,15].

The existing literature advocates an annual 2-h BLS and AED training from the age of 12 [16,17]. Unfortunately, they are difficult to implement in schools due to their operation. For example, in Spain, classes last 50 min each session; therefore, this implementation causes classes to come to a standstill, and many teachers are unwilling [18]. To implement this knowledge, there are numerous methods of teaching BLS to schoolchildren, in whom it was shown that 30-min BLS courses with mannequins were as effective as classic 2-h courses [19]. The flipped classroom methodology has been explored little in this regard. This teaching method seeks to maximise attention to the fundamental aspects of each subject, and thanks to it, the learning of practical skills in CPR and AED can be implemented within school hours since students work on theoretical content at home, and in the classroom, the focus is on practical skills, which are considered the key points [20]. With the use of the flipped classroom, it is intended to use a method that allows a large number of schoolchildren to be taught in a short time and prevents what has been learned from being forgotten as soon as possible [21]. For this reason, the European Resuscitation Council (ERC) recommends the use of virtual learning environments for e-learning of prior skills as part of a blended learning approach or for autonomous learning options [22].

The aim of this research was to compare the flipped classroom with traditional teaching and to explore the medium- and long-term retention of knowledge and practical skills learned among secondary schoolchildren after a CPR and AED course.

## 2. Materials and Methods

### 2.1. Study Design

A quasi-experimental design including post-test measurements [23] with follow-up at 6 and 12 months and a control group was used to perform this research.

### 2.2. Participants

Two secondary schools in northwestern Spain were invited to participate in the research. As an inclusion criterion, it was proposed that only students who did not have a physical or mental disability that prevented them from learning and performing basic CPR manoeuvres and use of the AED in the 1st and 3rd years of secondary education would participate in this research. The schoolchildren who correctly answered the questions regarding the sequence of action for a person with possible cardiac arrest and the correct order of application of an effective shock were rejected from the investigation. Schoolchildren who did not participate in the entire process or were admitted to the educational centre after the start of the study were excluded. In addition to this, the informed consent of their parents or legal guardians was necessary for participation in this study.

### 2.3. Tools and Measurements

The following tools were used in the research’s development, and the variables mentioned below were gathered:

#### 2.3.1. Ad Hoc Questionnaire

The ad hoc questionnaire used was made up of questions related to (1) the socio-demographic data of the schoolchildren (i.e., name and surname, age, and sex, among others); (2) previous training received in BLS (yes/no); (3) sequence of action for a person with possible cardiac arrest (1. scene security; 2. consciousness assessment; 3. breathing assessment; 4. emergency medical service (EMS) call; 5. position of the hands and initiation of external chest compressions); and (4) correct order of AED application (1. turn on, 2. apply patches, 3. insert patch connector, 4. follow instructions, 5. shock). Questions (3) and (4) were scored as correct or incorrect according to the answers and the order given by the schoolchildren since these answers were out of order and they had to number them in the correct order of execution.

#### 2.3.2. Anthropometric Data

The weight and height of each schoolchild were assessed barefoot and in comfortable clothing using a scale and a stadiometer, respectively. The body mass index (BMI) was calculated using the formula weight (Kg)/(height (m))^2^. The space used for this part of the research was cosy and allowed for the schoolchildren participants’ privacy. The room was occupied by the researchers and one person from the educational centre’s management staff for each gender. In both schools, the measurements were conducted uniformly and in accordance with the standard methods.

#### 2.3.3. CPR Data Collected

The Laerdal Resusci Anne CPR mannequin with SkillReporter software version 2.4 (Laerdal Medical AS, Stavanger, Norway) was used to perform CPR and collect data. It was exclusively programmed for chest compressions.

The variables related to the quality of CPR execution collected were % total quality of hands-only CPR (75–100%); total compressions in 1 min (100–120) and 2 min (200–240); % of correct hand positioning during compressions (100%); compression depth (50–60 mm); and % of compressions with complete re-expansion (100%). Variables were categorised as achieved/not achieved.

Prior to performing the external cardiac compressions, the researchers used a checklist to record whether the schoolchildren executed the following actions: scene security, consciousness assessment, breathing assessment, emergency medical system (EMS) call, positioning of the hands, and initiation of external chest compressions.

#### 2.3.4. AED Data Collection

The source of instruction for using an AED was the Laerdal AED trainer, a simulation of the HeartStart FR2+ Philips Defibrillator. The following variables were collected to record the use of the AED: 1. effective shock; 2. execution safety; 3. quality shock; 4. errors made; and 5. time to give an effective shock. An effective shock was considered one for which no errors were made that altered the target of the shock (i.e., chest patch below the midline of the heart; rib patch below the midline of the heart; delivering a shock without placing the patches; shocking without pasting the patches, or misplaced patches). Execution safety was considered if the schoolchildren were not in contact with the mannequin at the time of the shock. A quality shock was considered one for which no errors were made, the shock was performed safely, and the execution order (1. power on; 2. apply patches; 3. insert the patch connector; 4. follow instructions; and 5. shock) was correct.

### 2.4. Procedure

To carry out this research, the management of the 2 educational centres was contacted; the objective of the study was explained; and, later, a letter was sent to the parents and/or legal guardians explaining the objective, purpose, design, and procedure of the study (data collection, analysis methods, and their subsequent use), in addition to the declaration of confidentiality, voluntary participation, and the possibility of withdrawing the schoolchildren from the study at any time they wished.

The required sociodemographic data (age, sex, height, and weight) were recorded once the parents’ signed informed consent was obtained. In order to assist the construction of the training programmes in the control group (CG) (traditional training programme) and the experimental group (EG) (flipped classroom training programme), the schoolchildren were then randomised by natural groups (belonging to the same group-classroom and school). An ad hoc questionnaire was presented to the participating schoolchildren before the training programmes to ascertain their background knowledge of BLS.

A 50-min theoretical–practical course with instructor-led training in BLS and AED was provided to the CG. During this time, focus was placed on the value of performing hands-only CPR without pausing and using the AED in accordance with the guidelines set forth by the European Resuscitation Council (ERC) [24]. Assuring the victim’s safety in the area, identifying unconsciousness, opening the airway and controlling breathing, raising the alarm for emergency medical services, performing chest compressions, and starting an AED were all practised during the practical portion, which lasted 10 min and had a ratio of one monitor, one CPR mannequin, and one AED for every two schoolchildren (hands-only CPR for was performed for 2 min, and the partner applied the AED). Each schoolchild underwent this cycle twice.

The EG was given the same ad hoc questionnaire as the CG but through the virtual classroom in the physical education subject of each educational centre. This questionnaire had to be answered to gain access to 2 short videos; one of external cardiac massage of compressions only, lasting 3 min and 20 s (https://www.youtube.com/watch?v=ZQdwoRf-TLg (accessed on 17 October 2022)), and another on the use of AEDs, lasting 3 min 57 s (https://www.youtube.com/watch?v=6W4zbqWWDs20 (accessed on 17 October 2022).; both have been used in other similar studies [9,25,26]. The schoolchildren were able to watch the videos as many times as they wanted. The day after this task, the schoolchildren received the same practical part as the CG under the same conditions at school.

Following training, each schoolchild was led in solitude to a room with a simulated situation set up, including an AED and a Laerdal Resusci Anne Q-CPR mannequin set to compressions-only mode. Each schoolchild was instructed to behave in accordance with their retention of the training process. The parameters started to be recorded when the schoolchildren started performing hands-only CPR. The schoolchildren were instructed to use the AED on the mannequin’s bare chest after the 2 min of external cardiac compressions, recording any potential mistakes in its use and the time it took to deliver an effective shock. The interval between when they picked up the AED and when they activated the shock button was recorded. Two BLS experts evaluated the entire process in real-time by following the checklists.

The entire procedure was carried out in compliance with the Declaration of Helsinki and the most recent international standards for cardiopulmonary resuscitation [24]. The University of Santiago de Compostela’s Ethics Committee gave their approval of the research protocol.

### 2.5. Data Analysis

Quantitative variables were expressed as measures of central tendency (means and standard deviation), and categorical variables were expressed as frequencies and percentages, as appropriate. The *t*-test for independent samples was used before the application of the training programs to evaluate the differences between the control (CG) and experimental (EG) groups in terms of age, weight, height, and body mass Index. To check whether the previous training received, the sequence of action in terms of CPR and AED, and gender were similar in both groups, the chi-square test was used. After the training process was applied in both groups, to evaluate the differences between groups (CG vs. EG) in terms of the sequence of action for a person with possible cardiac arrest, as well as the achievement or not of quality external cardiac compression parameters and the application of the AED in terms of effective discharge, performance safety, and objective quality, the chi-square test with Cramer’s V statistic was used at each of the time points (post-training, 6 months after training, and 12 months after training). The independent samples *t*-test was used to establish the differences in the times of application of an effective shock. Cohen’s d statistic was used to establish statistical power (0.2 small; 0.5 medium; >0.8 large). SPSS software (SPSS v.25, IBM Corporation, New York, NY, USA) was used for all statistical analyses. The level of significance was set at *p* > 0.05.

## 3. Results

Initially, 309 schoolchildren were included in the study; however, 49 were excluded for completing the initial questionnaire’s CPR and AED action sequence questions properly. Finally, a total of 260 schoolchildren aged 12–14 years (*M* = 12.79; *SD* = 1.03) participated, with a mean weight of 54.96 kg (±12.22) and a mean height of 162.76 cm (±9.42). Of these 260 schoolchildren, 117 (45.0%) were girls and 143 (55.0%) were boys.

**Figure 1:**
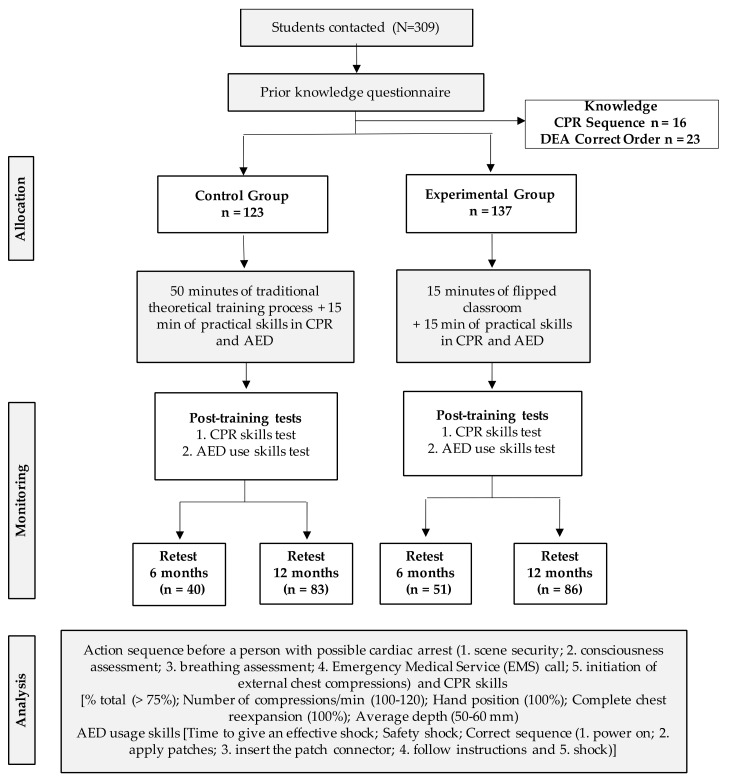
Research flowchart.

According to the natural groups by course and class to which they belonged, the schoolchildren participants were divided into the CG (traditional training, 123 schoolchildren) and the EG (flipped classroom training, 137 schoolchildren). A total of 91 schoolchildren participants (CG = 40 and EG = 51) were assigned to re-test after six months of training, and 169 schoolchildren participants (CG = 83 and EG = 86) were assigned to re-test after twelve months, on the basis of the availability of educational centres (Figure 1).

### 3.1. Baseline Characteristics

The initial characteristics of both groups appear in Table 1. The data indicate that there were no differences in sociodemographic variables between groups (CG and EG).

### 3.2. Results Regarding the Sequence of Action in the Event of a Possible Cardiac Arrest

The results for the CG and EG groups at the three time points analysed (post-training, 6 months, and 12 months) are shown in Table 2. At both the post-training time point and 6 months after it, no statistically significant differences were found between the CG and the EG in any of the variables studied. At 12 months after training, statistically significant differences were only found in the action of calling the emergency number 112 (*p* = 0.034; Cramer’s V = 0.163, *p* = 0.034), in favour of the CG (97.6% vs. 89.5%).

### 3.3. CPR Skills

Data regarding the quality of external cardiac compression skills are reflected in Table 2. Statistically significant differences were found between the two groups (CG vs. EG) after training in the number of schoolchildren who achieved 100% correct hand positioning during external cardiac compressions; 45.0% of the EG schoolchildren managed to accomplish this, compared with 29.3% of the CG schoolchildren (X^2^ = 6.450; *p* = 0.011, Cramer’s V = 0.158; *p* = 0.011). In addition to this, 101 schoolchildren (73.7%) in the EG managed to perform the correct depth range (50–60 mm) compared with 63 schoolchildren (51.2%) in the CG (X^2^ = 14.092; *p >* 0.001, Cramer’s V = 0.233; *p* = 0.001). Regarding compressions with complete re-expansion (100%), statistically significant differences were found in favour of the CG versus the EG (48.0% vs. 34.3%) (X^2^ = 5.009; *p* = 0.025, Cramer’s V = 0.139; *p* = 0.025). At 6 and 12 months after training, the statistically significant differences disappeared, and similar results were obtained in both groups (Table 2).

### 3.4. AED Application

The results regarding the application of an effective shock with the AED after training were 94.3% for the CG and 96.4% for the EG, and they were 97.5% for the CG and 98.0% for the EG at 6 months (Table 2). At 12 months, this percentage decreased in both groups (CG vs. EG) compared with the post-training time point and at 6 months. No statistically significant differences were found regarding this variable between the two training groups (CG vs. EG) at any of the three time points (post-training, 6 months, and 12 months).

Regarding the safety in the application of the AED, there were no statistically significant differences between the CG and the EG after training or at any of the three moments (post-training, 6 months, and 12 months) since in 100% of some occasions and close to 100% of others, the schoolchildren discharged the shock without touching the mannequin.

Regarding the performance of the shock without any error, the percentages were again similar in both training groups (CG vs. EG), with no statistically significant differences at the three time points (post-training, 6 months, and 12 months). However, the number of schoolchildren who applied the AED shock without any errors decreased in both groups when the time points were compared with each other (post-training vs. 6 months vs. 12 months).

The mean times of the schoolchildren who managed to perform an effective shock with the AED were significantly better in the EG after training (*p* = 0.013; Cohen’s d = 0.328). These times decreased at 6 months of training compared with post-training and subsequently increased at 12 months. Even so, the times obtained at 12 months compared with the post-training times were still lower, although the percentage of schoolchildren who were capable of applying an effective shock decreased.

## 4. Discussion

The objective of this research was to study the feasibility of teaching CPR and AED use with flipped-classroom methodology (EG) compared with a traditional teaching method and to explore the medium- and long-term retention of knowledge and practical skills among secondary schoolchildren, considered a target population in the position “kids save lives” promoted by the ERC [27].

The results obtained regarding the knowledge acquired after the application of the training methods indicate that both produced an increase in the knowledge and skills of schoolchildren [3,28,29,30]. Despite the fact that schoolchildren were capable of activating the chain of survival and starting CPR, they did so without sufficient quality, as observed in previous studies involving students of ages close to those in our study [31].

### 4.1. BLS Action Sequence

The BLS action sequence, as in previous studies [17,31], was followed by more than half of the schoolchildren except for scene safety. This is probably due to this study’s use of a simulated scenario with a mannequin in an environment, such as the one used (school), that may give false security. In this type of study and environment, schoolchildren do not pay as much attention to this aspect as if it were a real scenario with a human victim [32]. In this regard, it must be taken into account that the follow-up and application of the BLS steps according to the recommendations [24,33], are not easy to achieve [34].

Despite this, both training processes resulted in a large number of schoolchildren being able to follow the steps of this action sequence without significant differences between the CG and the EG [29]. This ability was maintained at 6 and 12 months, except for the action of calling the emergency services once the victim’s absence of breathing is detected, which was followed by a higher percentage of students who received the traditional training method. These findings are relevant as the ability to follow these steps in the face of a person experiencing out-of-hospital cardiac arrest is critically important [22], especially if we take into account that performing the steps correctly is not an easy task, even for a trained adult [35].

### 4.2. Hands-Only CPR Skills

Regarding the quality of the skills in performing CPR [24], approximately half of the participating schoolchildren in both the CG and the EG were able to execute quality CPR [36] and retained these skills throughout time [29,37] at 6 and 12 months [18]. However, the ability to perform the correct compression depth was the most difficult for many of the participants in this study [30]. There was no difference in CPR skills between the CG and the EG, as evidenced by a recent systematic review and meta-analysis [38].

In more detail, the performance of quality external cardiac compressions is closely related to the anthropometry of schoolchildren [39,40]. Thus, the EG obtained better percentages than the CG, despite the fact that there were no differences in the anthropometric characteristics in both training groups [41,42]. The EG also obtained better percentages in the correct positioning of the hands during external cardiac compressions and at the correct mean depth. These differences were reduced at 6 months as well as at 12 months [32,43,44].

The percentage of compressions with complete re-expansion is also an indication of the quality of the external cardiac compressions so that the chest is fully released [24,33]. When analysing this variable, it was observed that half of the schoolchildren achieved 100% in both groups [24,33], and this percentage increased after 6 and 12 months of training. This could be because the percentage of schoolchildren achieving adequate depth is lower, and consequently, re-expansion of the thorax is easier to achieve [45].

The adequate rate of compressions, which could be internalised by the feedback given by the mannequin during training [46], depends not only on the anthropometry of the schoolchildren [47] but also on the level of development and maturity of the motor skills [48]. In this study, the percentage of the rhythm was similar in both groups and did not reach 50% after training. At 6 and 12 months, the percentage of schoolchildren who maintained this range of compressions per minute increased slightly, but in no case did it reach 35% of the total.

### 4.3. AED Application

The percentages of performing the sequence in the correct order without committing any error in the application of the AED were low in both groups, and this percentage decreased at 6 and 12 months after the training. These findings are in line with what was proposed by the ERC on education for resuscitation [22], which indicates that practical skills in any activity need regular training for adequate performance, and in the case of AED, this seems to be between 6 and 12 months.

Regarding the simulation of an effective shock with the AED, and after receiving the training, the percentage of schoolchildren who managed to perform an effective shock was almost 100% in both groups (CG and EG), a percentage that was maintained in those schoolchildren evaluated at 6 months [15]. However, this percentage decreased in the schoolchildren evaluated at 12 months, so refreshment of this skill should be made before this time elapses [22].

Regarding the safety in the simulation of the application of a shock with the AED, almost 100% performed it correctly in both groups (CG vs. EG) and at all time points (post-training, 6 months, and 12 months) [4].

Lastly, when evaluating the time spent applying an effective shock with the AED, the schoolchildren who were able to apply it did so in just over 60 s, a time similar to that in previous studies with a population of similar age [15,49]. These results may suggest the importance that schoolchildren give to the time factor in the case of cardiac arrest and early defibrillation [49,50].

The limitations found were (1) the assignment to the control (CG) and experimental group (EG) was not random and (2) the study involved a simulation on a mannequin, and therefore, the results must be taken with caution since we do not know how the schoolchildren would react in a real situation.

## 5. Conclusions

The results obtained in this study highlight that training through the flipped classroom is feasible and can be implemented in the normal development of classes in schools without interrupting their normal development or modifying the time structure of the didactic sessions (50 min). In addition, it allows the implementation of the teaching of the sequence of action for a person with possible cardiac arrest, the learning of quality cardiac compressions, and the correct execution of the steps to follow to apply an effective shock with an AED with the same results as traditional teaching but in less training time, according to the standards recommended by international institutions [24], so that teaching can be focused on practical skills.

For this reason, it is considered that training courses in BLS with mixed methodology should be promoted, in which the theoretical part can be worked on by secondary schoolchildren at home, and in this way, more emphasis can be placed on practical training, in which secondary schoolchildren obtain the amount optimal training necessary for proper performance of BLS and AED skills. Even so, it is recommended to refresh the action sequence and CPR before 6 months and skills in the application of an effective shock between 6 and 12 months.

## Figures and Tables

**Table 1:** Sample baseline characteristics.

Variable	Control Group (*n* = 123)	Experimental Group (*n* = 137)	*p*-Value
Age (years)	12.84 ± 1.02	12.74 ± 1.03	0.364
Gender (male/female)	67/56	76/61	0.437
Weight (kg)	54.88 ± 12.44	55.03 ± 12.06	0.921
Height (cm)	162.76 ± 9.74	162.76 ± 9.07	0.995
Body mass index (kg·m^−2^)	20.60 ± 1.02	20.67 ± 1.03	0.880
Previous training received (yes/no)	97/26	107/30	0.882
CPR action sequence (correct/incorrect)	123/0	137/0	-
AED action sequence (correct/incorrect)	123/0	137/0	-

Note. Data are presented as mean ± standard deviation or mean and frequencies.

**Table 2:** Evaluation of CPR and AED skills after training (CG vs. EG) at 6 and 12 months.

		Post-Training (*n* = 260)		6 Months Post-Training (*n* = 91)		12 Months Post-Training (*n* = 169)	
		CG (*n* = 123)	EG (*n* = 137)	CG (*n* = 40)	EG (*n* = 51)	CG (*n* = 83)	EG (*n* = 86)
Scene security	No	52 (42.3%)	42 (30.7%)	5 (12.5%)	6 (11.8%)	8 (9.6%)	3 (3.5%)
	Yes	71 (57.7%)	95 (69.3%)	35 (87.5%)	45 (88.2%)	75 (90.4%)	83 (96.5%)
	*p*-value	0.052		0.915		0.105	
Consciousness assessment	No	10 (8.2%)	14 (10.2%)	1 (2.5%)	5 (9.8%)	1 (1.2%)	5 (5.8%)
	Yes	113 (91.8%)	123 (89.8%)	39 (97.5%)	46 (90.2%)	83/98.8%)	81 (94.2%)
	*p*-value	0.561		0.163		0.105	
Breathing assessment (open airway and check breathing)	No	3 (2.4%)	4 (2.9%)	1 (2.5%)	2 (3.9%)	2 (2.4%)	2 (2.3%)
	Yes	120 (97.6%)	133 (97.28%)	39 (97.5%)	29 (96.1%)	81 (97.6%)	84 (97.7%)
	*p*-value	0.811		0.706		0.971	
Emergency call	No	19 (15.4%)	20 (14.6%)	4 (10.0%)	3 (5.9%)	2 (2.4%)	9 (10.5%)
	Yes	104 (84.6%)	117 (85.4%)	36 (90.0%)	48 (94.1%)	81 (97.6%)	77 (89.5%)
	*p*-value	0.848		0.464		**0.034**	
Percentage of total compressions	75–100	48 (39.0%)	57 (41.6%)	22 (55.0%)	22 (43.1%)	40 (48.2%)	46 (53.5%)
	Oher	75 (61.0%)	80 (58.4%)	18 (45.06%)	29 (56.9%)	43 (51.8%)	40 (46.5%)
	*p*-value	0.672		0.296		0.540	
Total compressions in 1 min	100–120	24 (19.5%)	40 (29.2%)	12 (30.0%)	15 (31.4%)	36 (43.4%)	32 (37.2%)
	Other	99 (80.5%)	97 (70.8%)	28 (70.0%)	35 (68.6%)	47 (56.6%)	54 (62.8%)
	*p*-value	0.070		0.888		0.436	
Hand position during compression	100%	36 (29.3%)	61 (44.5%)	31 (77.5%)	33 (64.7%)	61 (73.5%)	70 (81.4%)
	Other	87 (70.7%)	76 (55.5%)	9 (22.5%)	18 (35.3%)	22 (26.5%)	16 (18.6%)
	*p*-value	**0.011**		0.249		0.270	
Compression depth	50–60 mm	63 (51.2%)	101 (73.7%)	24 (60.0%)	27 (52.9%)	46 (55.4%)	56 (65.1%)
	Other	60 (48.8%)	36 (26.3%)	16 (40.0%)	24 (47.1%)	37 (44.6%)	30 (34.9%)
	*p*-value	**<0.001**		0.530		0.212	
Compressions with full re-expansion	100%	59 (48.0%)	47 (34.3%)	20 (50.0%)	28 (54.9%)	43 (51.8%)	39 (45.3%)
	Other	64 (52.0%)	90 (65.7%)	20 (50.0%)	23 (45.1%)	40 (48.2%)	47 (54.7%)
	*p*-value	**0.025**		0.677		0.443	
AED application with effective discharge	No	7 (5.7%)	5 (3.6%)	11 (27.5%)	11 (21.6%)	23 (27.7%)	27 (31.4%)
	Yes	116 (94.3%)	132 (96.4%)	39 (97.5%)	50 (98.0%)	60 (72.3%)	59 (68.6%)
	*p*-value	0.313		0.623		0.617	
AED application safely	No	1 (0.8%)	0 (0%)	0 (0%)	0 (0%)	0 (0%)	3 (3.5%)
	Yes	122 (99.2%)	137 (100%)	40 (100%)	51 (100%)	83 (100%)	83 (96.5%)
	*p*-value	0.473		-		0.246	
AED application without any errors	No	47 (38.2%)	41 (29.9%)	23 (57.5%)	40 (78.4%)	62 (74.7%)	61 (49.6%)
	Yes	76 (61.8%)	96 (70.1%)	17 (42.5%)	11 (21.6%)	21 (25.3%)	25 (29.1%)
	*p*-value	0.101		0.041		0.608	
Average time to apply an effective shock	*M* (*SD*)	69.19 (10.83)	66.15 (7.62)	57.49 (8.60)	54.74 (9.19)	59.08 (12.55)	59.79 (9.26)
	*p*-value	**0.013**		0.213		0.725	

Note: *M*: Mean; *SD*: Standard deviation; CG: Control Group (traditional training); EG: Experimental Group (flipped classroom training).

## Data Availability

The data presented in this study are not available in accordance with Regulation (EU) of the European Parliament and of the Council 2016/679 of 27 April 2016 regarding the protection of natural persons with regard to the processing of personal data and the free circulation of these data (RGPD).
